# Altered Neural Response Induced by Central-Fatigue in the Cortical Area During High-Intensity Interval Pedaling

**DOI:** 10.32598/BCN.9.10.440

**Published:** 2019-11-01

**Authors:** Mehrangiz Ghorbani, Farshad Ghazalian, Khosrow Ebrahim, Hossein Abednatanzi

**Affiliations:** 1. Department of Physical Education and Sport Sciences, Faculty of Humanities and Social Sciences, Science and Research Branch, Islamic Azad University, Tehran, Iran.; 2. Department of Physical Education and Sport Sciences, Bijar Branch, Islamic Azad University, Bijar, Iran.; 3. Department of Physical Education and Sport Sciences, Faculty of Humanities, Shahid Beheshti University, Tehran, Iran.

**Keywords:** Fatigue, Electroencephalography, High-intensity interval training

## Abstract

**Introduction:**

The central-governor model explains the mechanism of endurance exercise-induced central fatigue, but high-intensity exercise-induced central fatigue has not been investigated yet. This study aimed to research how central fatigue during high-intensity intermittent pedaling alters the neural response, which results in Electroencephalography (EEG) recordings.

**Methods:**

We assessed neural response by measuring the alternation of brainwave spectral power during an intermittent high-intensity 60-minute exercise on an ergometer cycle. The cadences were changed every 10 minutes according to intermittent pattern altering (90-120-60-120-60-90 rpm). EEG was used to analyze altering brain function. Heart Rate (HR), Blood Lactate (BL), and Rating of Perceived Exertion (RPE) were measured after the change in cadences.

**Results:**

HR, BL, and RPE increased at a cadence of 120 rpm compared with 60 rpm on the ergometer cycle. The spectral power of EEG, according to cadence × brainwaves, significantly increased (P<0.01) in the alpha and beta frequency ranges with a change in cadences between 60 rpm and 120 rpm. The spectral power of the EEG significantly increased (P<0.01) over the whole frequency range from rest to warming (theta: 251%, alpha: 165%, beta: 145%) and significantly reduced in theta, alpha, and beta (theta: 176%, alpha: 142%, beta: 77%) (P≤0.01).

**Conclusion:**

High-intensity exercises (90 and 120 cadences) increased brain function, regardless of fatigue occurrence. High-intensity interval training (HIIT) led to altering the neural response. It would be required to investigate the usefulness of HIIT to treat some of the psychotic disorders.

## Highlights

- Central nervous system control in cardiac function largely depends on the intensity of training determined by a change of cadence in the ergometer cycle.
- High-intensity interval training increases brain neural efficiency.
- Exercise at high intensities improves metabolic function, cardiac function, and breathing capacity.
- The brain’s role with attention to its neural response in changing position is crucial. We could emphasize it by planning appropriate training to improve the function of the brain.

## Plain Language Summary

The human brain is a complex part of the body that defines us as human. Sport and exercise are not food but feed the brain and other organs. The brain adjusts for varying commands by changing its functional and structural characteristics, which result in learning and acquiring new skills (in this article, the function is the high-intensity interval pedaling). We review the variety of brain activities induced by fatigue because of two main causes; 1. local muscular fatigue and 2. mental (central) fatigue. While the local muscular system can recover, the fatigue of the central nervous system can take much longer. High-intensity interval training has the largest impact on the central nervous and cognitive systems. These effects are interpreted as their behavioral outcomes.

## 1. Introduction

Central fatigue is an unfrequented phenomenon expressed in both populations of healthy (during physical activity) and non-healthy people. In healthy people, fatigue is a predicted mechanism after a prolonged and intensive activity that is recovered by rest (Gandevia, 2001; Kluger, Krupp, & Enoka, 2013). Fatigue is experienced in both physical and perceptual aspects (Kluger et al., 2013; Enoka & Stuart, 1992; Taylor & Gandevia, 2008). “Central” and “peripheral” fatigue should also be differentiated. Central fatigue is known as the failure of the performance because of the improper function of the central nervous system. Prolonged or intensive exercise leads to central fatigue (Weavil, Sidhu, Mangum, Richardson, & Amann, 2016). High-intensity interval training (HIIT) improves function and mental health in patients with cardiopulmonary diseases by increasing brain-derived neurotrophic factor and improving neural plasticity in the rats (Luo et al., 2019). Neural plasticity is an expected property of neurons that is vital in adaptation to new situations (Assenza, 2015).

Many researchers have already investigated physical and physiological adaptations, especially the cardiovascular and respiratory systems (Weavil et al., 2016; Taylor, Amann, Duchateau, Meeusen, & Rice, 2016). Vast research has been organized on the ergometer cycle performance from recreation to the expert levels in elite athletes, especially among endurance athletes (Faria, Parker, & Faria, 2005). However, changes in the central nervous system and brain activity, as well as changes in the behavioral pattern of neurons during exercise were not considered during high-intensity interval exercises. HIIT could be used to raise sports performance and brain function in men and women alike (Coetsee & Terblanche, 2017; Chun Kao, Westfall, Soneson, Gurd, & Hillman, 2017). Brain activity regulates the homeostasis in athletes to delay fatigue and achieve optimal performance during endurance competitions (Noakes, 2011; Skorski & Abbiss, 2017), but recovery strategies in HIIT induce fatigue and involve organisms rather than modulating homeostasis. Active recovery increased the aspects of central rather than muscle recovery (Rattray, Argus, Martin, Northey, & Driller, 2015; Giboin, Amiri, Bertschinger, & Gruber, 2018; Gruet et al., 2014). The study of brain activity by using electroencephalography (EEG) recording has many advantages in sports and training (Skorski & Abbiss, 2017). All of our thoughts, emotions, and behaviors are due to the communication between neurons within our brains. Brainwaves are produced by synchronized electrical pulses from masses of neurons communicating with each other. Neural response patterns in brain activity in a high-intensity intermittent protocol, which is useful to increase cardiac function, provides useful information to different parts of the brain to obtain the general idea of treating many diseases (Nobrega et al., 2014).

Many scientists have investigated the changes in brain activity caused by cycling (Comani et al., 2013). Brain activity is changed by altering functional properties in response to demands, using the central governor model and the homeostasis phenomenon (Inzlicht & Marcora, 2016; Noakes, 1997; Noakes, 2000; Noakes, 2012). The evaluation of the patterns of neural response of the cerebral cortex (neuroplasticity) (Budde, Wegner, Soya, Rehage, & McMorris, 2016) during intervals with fatigue contributes to a new insight into functional connectivity between different parts of brain activity in HIIT (Taylor & Gandevia, 2008; Taylor, et al., 2016; Comani et al., 2013; Gotshall, Bauer, & Fahrner, 1996). We hypothesized in the present study that neural response patterns induced by the central fatigue in the cortical area are changed by increasing the intensity of training. Therefore, this research aimed to evaluate the effects of central fatigue during high-intensity pedaling with intermittent changes on brain activity. These changes are done by altering functional properties, which results in spectral power frequencies of brainwaves.

## 2. Methods

### 2.1. Study Participants

Fourteen active sprint cyclist women (with the Mean±SD age of 25.9±3.8 years, height of 170±1.6 cm, and weight of 62.4±2.2 kg) were selected according to the following inclusion criterion of 4–6 h or 200–240 km (15–40 km/h) training background on the flat surface per week (Ludyga, Gronwald, & Hottenrott, 2015). After the researcher explained the study design, potential risks, and benefits, each participant signed the informed consent form to participate in the study. The exclusion criteria included any cardiovascular and respiratory, metabolic, psychiatric diseases, or orthopedic trauma that could restrict the training. The participants were informed that they were free to leave the study whenever they want. The Ethics Committee of the Medical Faculty of the Azad University of Science and Research Branch of Tehran confirmed this study under the number of IR.IAU.PS.REC. 1397-115, according to the Declaration of Helsinki.

### 2.2. Testing protocols

To determine the anaerobic capacity of the participants, they accomplished the following high-intensity performance test on an ergometer cycle (Monark 894E, Anaerobic Wingate testing): start, 110 W; increase, 10 W; and duration, 4 minutes. The test evaluated anaerobic capacity by a spirometry system (cortex, Meta max 3b, Germany). The participants executed this stage of the test to the failure of power output, and voluntary fatigue occurred. After completing the test, the maximum oxygen consumption (VO2 _max_ ) and maximum load (P _max_ ) are determined. Aerobic and anaerobic blood lactate (BL), as well as maximum steady-state lactate concentration threshold ([mmol. l ^−1^ ]. [Watt] ^−1^ ) were assessed (Mann, Lamberts, & Lambert, 2013). BL was measured by an enzymatic-amperometric method (Lin, Chen, & Lin, 2018) in 10 μL blood sampled from the earlobe and was analyzed via WinLactat 4.1.0.1 (XE Version, German).

After a week and 3 hours, a meal, the participants performed a 60-minute pedaling test in different cadences with 90% VO2 _max_ . The staging test included standard warm-up (10 minutes with 100 W), the main protocol (6 ten-minute steps), and cool-down (10 minutes at 100 W). The cadence intermittent (90-120-60-120-60-90-60 rpm) changed every 10 minutes during the exercise. The rating of perceived exertion (RPE) was used to assess the intensity of the training interval, using the Borg scale (Borg, 1998). The HR on EEG setup, BL, and RPE were recorded at the end of each 10 minutes. The rest were recorded after the warm-up, after every 10-minute cadence, and after the cool-down (Amann, 2011; Gronwald, Hoos, Ludyga, & Hottenrottd, 2018).

### 2.3. EEG recordings and analysis

Electroencephalography (64-Channel QuickAmp-EEG system, Brain Products, Germany) was used in continuous brain cortical activity recording. The changes in brainwave signals were symmetrically recorded by 21 active surface electrodes (Brain Products, EasyCap, Germany; Fp1, Fp2, F7, F8, F3, Fz, F4, FC7, FC8, T7, T8, C3, CZ, C4, CP1, P7, P3, P4, PZ, P8, CP2, 10:20 Fixed EEG flexible caps [EasyCAP, Germany]) (Klem, Lüders, Jasper, & Elger, 1999; Jung, Makeig, Bell, & Sejnowski, 1998). We implemented the active Ag/AgCl electrodes in an active circuitry, ActiShield, a system that allowed the recording of high contact resistance to 60 kΩ. The electrode impedances were controlled by EasyCAP Drivers 6.10.70.001 Software version 1.2.5.3 (Brain Products, Germany), the signals were recorded by Brain Vision Recorder 1.03 (Brain Products, Germany), and the data were sampled at a frequency of 500 Hz. Matlab EEGLAB software was used for offline processing EEG raw data. EEG data were analyzed at rest, after warm-up, before each change in cadence, after cool-down, rest, and exercise. After editing, the data were filtered: high-pass, 1.5 Hz, octave ^−1^ 12 dB slope; low-pass, 50 Hz, octave ^−1^ 48 dB between 1–2 Hz and >50 Hz for removing muscle artifacts. Artifacts up to theta frequencies (4 Hz) and minimum frequencies of beta (30 Hz) were eliminated by frequency analysis and, then, a manual raw data inspection was carried out to identify the artifacts. Also, independent components’ analysis was calculated to remove artifacts from the main signals (Faria et al, 2005; Faria, Parker, & Faria, 2005). This control was fixed off-line and analyzed only by EEG sections without artifacts. After the edited markers, the signals were divided into 4 S-data-sets with a corrected baseline (Amann, 2011) at the last minute of each time point. Each participant and measuring time point 5 artifact-free segments were analyzed by the Fast-Fourier transform method (Maximum resolution, power in μV2, full-range use, hanging window, window length: 20%). The received frequency spectrum was divided into 3 frequencies: theta, 4–7 Hz; alpha, 8–15 Hz; and beta, 16–30 Hz. For further processing, the values were transferred to Microsoft Excel 2016. The analysis of 21 electrodes for each frequency range and time point were measured. The absolute values of spectral power as some changes from individual resting conditions (100%) was calculated to extract the effect of the different base EEG types (Faria et al, 2005).

### 2.4. Statistical analysis

The obtained data were analyzed in SPSS V. 24. The Kolmogorov-Smirnov test was performed to evaluate the normal distribution of data. In the case of a normal distribution, the difference between the time points (spectral power of EEG [%], HR [min ^−1^ ], BL [mmol. l ^−1^ ]) from analysis of variance test for repeated measurements, cadence×waves, and cadence×variables (of HR, BL) were calculated. For ordinal data (RPE), the Wilcoxon test was used. Different points of statistical significance were found out at P≤0.01 ( ^**^ ).

## 3. Results

**Figure 1.**
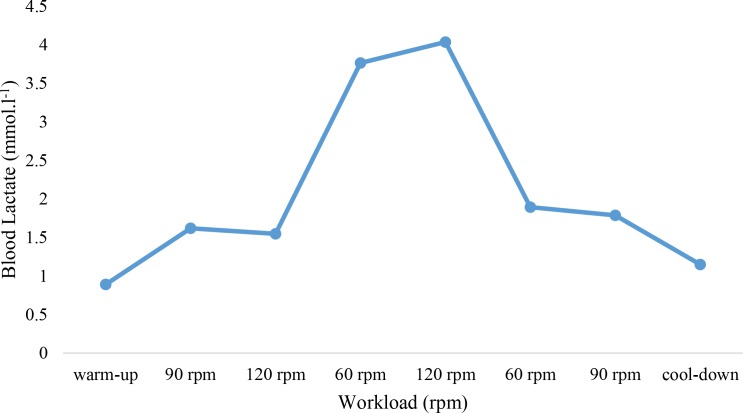
Blood lactate (mmol. l ^−1^ ) variability during warm-up, 6 states of changing cadences, and cool-down

**Figure 2.**
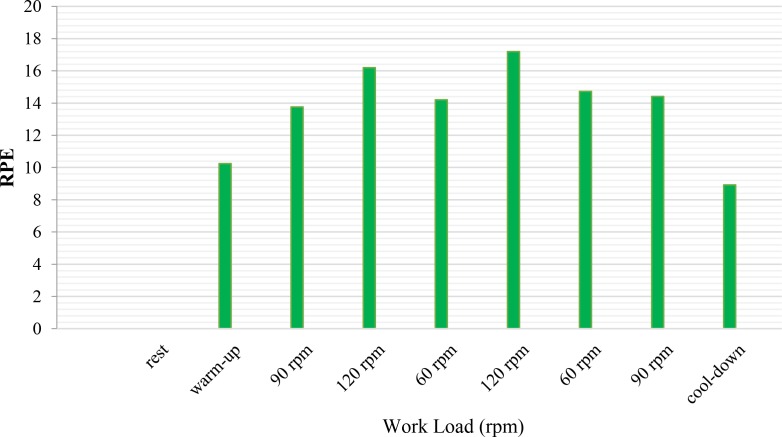
Rating of perceived exertion variability during warm-up, 6 states of changing cadences, and cool-down

The participants achieved a Mean±SD P _ma_ x of 308.92±20.61 (90% of the anaerobic lactate threshold: 224.71±19.00 W) and the VO2 _max_ of 40.43±3.67 mL· min ^−1^ · kg ^−1^ in ergometer cycle. The values of the heart rate (HR) and the BL during warm-up and at the beginning of the exercise test significantly increased (P<0.01). RPE significantly increased (P<0.01) at the beginning of the exercise test compared to the warm-up period. At 120 rpm, HR, BL, and RPE were significantly higher than 60 rpm. All physiological values significantly decreased (P<0.01) during the cool-down stage (Table 1, Figure 1 and Figure 2).

**Table 1.** Rating of perceived exertion, blood lactate, and heart rate during warm-up, 6 states of changing cadences, and cool-down

**Pedaling States**	**Variables**			

	**Cadence (rpm)**	**RPE (6–20)**	**Blood Lactate (mmol.l-1)**	**Heart Rate (min ^−1^)**
Warm-up	90	9.25±1.97	0.8±0. 4	118±8.67 ^**^
Workload 90 % of the LT	90	13.05±1.17 ^**^	1.5±0.57 ^**^	144.5±9.69 ^**^
	120	15.85 ±1.17 ^**^	3.5±1.12 ^**^	163.81±9.85 ^**^
	60	13.74±1.48 ^**^	1.57±0.57 ^**^	150.25±10.47 ^**^
	120	16.50±1.79 ^**^	3.5±1.16 ^**^	168.85±10.74 ^**^
	60	14.35±1.58 ^**^	1.8±0.45 ^**^	154.58±10.67 ^**^
	90	14.25±1.37	1.75±0.57	157.55±11.71 ^**^
Cool-down	60	8.31±1.85 ^**^	1.0±0.25 ^**^	118.65±8.57 ^**^
Rest	-	-	1.02±0.34 ^**^	62.55±8.77

SD. Standard Deviation; RPE. Rating of Perceived Exertion; RPM. Revolutions Per Minute; BL. Blood Lactate; HR. Heart Rate Data are presented as Mean±SD.

** Significant levels are at P<0.01.

**Figure 3.**
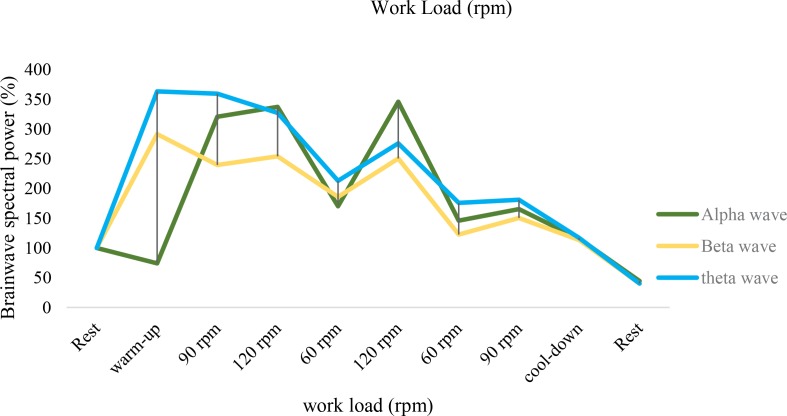
Percent variation in spectral power calculated over 21 electrodes compared to the rest situation (100%) and averaged over the full electroencephalography frequency dominant (4–30 Hz) of participants

The spectral EEG power was significant (P<0.01) for the entire rest to warm-up frequency ranges (theta: +251%, alpha: +182%, beta: +131%). The cadence changes are reflected in the spectral power of the EEG (Table 2 and Figure 1). By comparing the different cadences, the spectral power of the EEG (P<0.01), in theta, beta. The brain activity at the resting stage significantly decreased (theta: −67%, alpha: −58%, beta: −63%) in all cadences (P<0.01) compared with the beginning (90 rpm). According to the EEG brain map in the cadence of 120 rpm theta-waves, clear in all the brain (Figure 3).

**Table 2.** Variables of electroencephalography spectral power in rest, warm-up, 6 states of changing cadences, cool-down and, and rest

**Pedaling States**	**Variables**			

	**Cadence (rpm)**	**Theta (%)**	**Beta (%)**	**Alpha (%)**
Rest	-	100	100	100
Warm-up	90	350±160 ^**^	230±130 ^**^	282±200 ^**^
Workload at 90 % of the LT	90	345±194	230±110	308±284
	120	317±201	255±145	361±395
	60	200±133 ^**^	145±82 ^**^	171±112 ^**^
	120	260±175	210±166	335±455
	60	165±90 ^**^	112±57 ^**^	143±80
	90	170±85 ^**^	152±111 ^**^	150±85
Cool-down	60	147±114 ^**^	103±75 ^**^	114±78
Rest	-	35±22 ^**^	38±21 ^**^	41±22 ^**^

RPM. Revolutions Per Minute.

** Significant levels are at P<0.01. All data are shown via Mean±SD.

## 4. Discussion

The brain neural response decreases in a fatigue condition during pedaling. EEG recordings in different intensities, but at the same workload, showed a higher cadence pedaling frequency, associated with increased brain activity, HR, BL, and RPE. When recruiting muscle fibers increases, muscular coordination and higher cardiovascular function are needed to maintain a higher cadence (Enoka & Stuart, 1992; Taylor & Gandevia, 2008; Del Percio et al., 2009). BL increased in the state of high cadence (120 rpm) and decreased at a low cadence (60 rpm). These results support past results on how the training’s intensity changes affect the energy supply (Del Percio et al., 2009; CE, Rampichini, & Veicsteinas, 2009). In a steady-state workload, BL rises further at cadences from 100 rpm to 120 rpm (CE, et al., 2009) because of the enhanced HR caused by enhancing the cadence. Some investigators suggest that blood flow and, therefore, BL accumulation increases in the involved muscles (CE, et al., 2009). The consciousness of higher cadence exertion enhances with an increased HR and BL accumulation (CE, et al, 2009; Del Percio et al., 2009). Bailey showed a correlation between brain activity oscillations and physiological aspects of performance (Bailey, Hall, Folger, & Miller, 2008). The power spectral increase of the EEG has been clear in 120 rpm intensity. The brain activity should adapt to the performance intensity and power output in sports. Fatigue reduces brain activity. (Noakes, 2011; Weavil et al., 2016) also showed that brainwave oscillations reduced after a prolonged exhaustive exercise protocol displayed in the theta and alpha frequencies. Gronwald et al., performed the same research in cardiovascular regulations in exercise-induced fatigue by emphasizing the changes of cadences (Gronwald et al., 2018). The neural efficiency hypothesis was approved concerning the adaptation of the brain with the frequency of cycling with a lower level of beta activity in cyclists (Ludyga, Gronwald, & Hottenrottd, 2016). Ludyga also showed a reduction in brain activity after intensive ergometer cycling training. It was approved as a mechanism of fatigue to sustain homeostasis, which was explained in the central governor theory. To describe the fatigue mechanism, Noakes has proposed the central governor model for over two decades. This model introduces the unconscious mind of the brain in regulating the power output by modulating the force and applying the motor units to maintain homeostasis, which keeps the body from disastrous physiological disorders. In contrast, neural efficacy assumption depends on task demand and conscious awareness. The better adaptation of the brain activation product is because of the face to the challenges (Noakes, 2012). The neural efficiency hypothesis was characterized by adapting brain activation with functions. There is a correlation between high-cardiac output and increased BL accumulation during high intensities and, then, the perception of force exertion increased (Hagberg, Mullin, Giese, & Spitznagel, 1981).

Referring to the results, the variables such as BL and HR altered with the intermittent changes. Both central governor theory and the neural efficiency hypothesis are clear with the findings of this study. Meanwhile, any change in cadence results in significant changes in all variables, and homeostasis maintains according to the central governor model. These changes occurred because they were adaptive and changed by the constant workload. A more complex cognitive task is consistent with this task; so, carrying out to a long, intense exercise is needed to reduce the energy reserves of the individual (Inzlicht & Marcora, 2016). It was mentioned as self-control or inhibitory control, a term borrowed from psychology. Self-control is the ability to regulate one’s body requirements, emotions, thoughts, and behavior in the face of internal or external stimulations to achieve specific goals (DeLisi, 2014).

The beta brainwave is a fast activity; it is present when we are attentive, alert, engaged in decision-making, judgment, problem-solving, or focused on mental activity. This band was presented in the frontal, occipital, and temporal lobe in 120 rpm. The frontal lobe includes dopamine neurons. The dopaminergic pathways are related to attention, motivation, reward, planning, and short-run memory duties. Dopamine limits sensory information that arrived from the thalamus to the forebrain. The motivation was considered in sports and exercise. The theta brain state, where it is realized, everyone can create everything and change reality immediately. The theta band is displayed in the entire area of the brain in approximately 120 rpm. Theta band is powerful and should be considered the subconscious (mind condition between the conscious and the unconscious).

Fatigue induced by high-intensity intermittent pedaling disables the brain, but at high intensities (cadence of 120 rpm) were obtained at one session, with repeatability in the 8 interval steps of the high-intensity workload following a behavioral pattern.The results were obtained at one session, with repeatability in the 8 interval steps of the high-intensity workload following a behavioral pattern. With the onset of fatigue, the activity of the brain decreases, but with increasing intensity, the activity of the brain increases. Fatigue in relation to HIIT creates new behavioral patterns in response to various situations according to the spectral power of alpha, beta, and theta with attention to involving areas of the cortical brain.

## 5. Conclusion

Thus, the pattern of brain activity oscillations depends on exercise intensity, and individually-preferred cadence contributes to the positive response of the cognitive process (Brümmer, Schneider, Strüder, & Askew, 2011). The activity of the brain cortex is crucial to achieving performance and power output in exercises. The higher parts of the cortex process cognitive abilities and are responsible for tolerating pain and delaying the pleasure by self-control. HIIT improves cognitive processes in older people (Coetsee & Terblanche, 2017; Samuel et al., 2017). In conclusion, fatigue induced by high-intensity intermittent training leads to a strategy to maintain homeostasis, not a mechanism to survive to the extent that is mentioned by the central governor model.

## Ethical Considerations

### Compliance with ethical guidelines

All procedures were done following the Helsinki Declaration. The Ethics Committee of the Medical Faculty of Azad University of Science and Research Branch, Tehran (1397-115), approved the present research.
